# In Sickness and in Health: Erythrocyte Responses to Stress and Aging

**DOI:** 10.3390/ijms23136957

**Published:** 2022-06-23

**Authors:** Marianna H. Antonelou, Angelo D’Alessandro, Anastasios G. Kriebardis

**Affiliations:** 1 Department of Biology, School of Science, National and Kapodistrian University of Athens (NKUA), 15784 Athens, Greece; 2 Department of Biochemistry and Molecular Genetics, School of Medicine–Anschutz Medical Campus, University of Colorado, Aurora, CO 80045, USA; angelo.dalessandro@cuanschutz.edu; 3 Laboratory of Reliability and Quality Control in Laboratory Hematology (HemQcR), Department of Biomedical Sciences, School of Health & Welfare Sciences, University of West Attica (UniWA), 12243 Egaleo, Greece; akrieb@uniwa.gr

Mature red blood cells (RBC) are the most abundant host cell in our body. Despite their apparent simplicity and unconventional composition (e.g., lack of nuclei and organelles) RBC respond to circadian fluctuations, to endogenous and exogenous stresses, and to environmental changes and stimuli, such as exercise, hypoxia, inflammation, mechanical tension, pharmaceuticals, dietary factors, and oxidants. Exposure to such stressors modifies RBC shapes, surface topology and membrane integrity and deformability, by targeting a protein “hub” of pivotal structural and functional relevance to RBC. These stressors also reprogram redox and energy metabolism, promote sequestration of circulating chemokines, mitochondrial DNA or pathogens, and finally, modulate the RBC secretome. The latter includes proteins (especially hemoglobin—Hb), lipids and metabolites (e.g., ATP, lactate, ascorbate), either as (actively) transported molecules or encapsulated within extracellular vesicles (EVs) of small and medium size.

RBC aging is followed by modifications in cellular shape, volume, composition, metabolism, and abundance or post-translational modifications of several surface molecules, some of which generate senescence signals that trigger erythrophagocytosis. While numerous factors, including chronic and acute oxidative stress, have been shown to contribute to RBC elimination in vitro and in vivo, the etiological triggers of removal of senescent RBCs from circulation are still incompletely understood. This holds true not just in the context of physiological aging, but especially in response to pathologies that alter RBC capacity to deliver oxygen (e.g., thalassemia, sickle cell disease) or to counteract oxidant stress (e.g., glucose 6-phosphate dehydrogenase, G6PD). Beyond oxygen delivery, the utmost relevance of RBC to regulation of vascular tone, blood coagulation, and immunomodulation, in addition to pitfalls of iatrogenic interventions (e.g., storage at blood bank) that may harm and modulate RBC while making them available on demand, create new challenges in the field of transfusion medicine.

This Special Issue focused on cellular and molecular pathways that mediate RBC responses to aging and stressful conditions, and how activation of these pathways contributes to a spectrum of systemic phenotypes of clinical and sub-clinical relevance. The contributions included a selection of carefully designed original research studies and well-written, comprehensive review articles that converge in the complex crossroads between RBC phenotypic changes and stress responses in health and disease, in vivo and in vitro. Data and opinions reported here by outstanding colleagues in the field (Drs P. Buehler, P. Cabrales, P. Connes, D. Devine, N. Di Pietro, M. Prudent, K. Marzec, T. Nemkov, P. Rondeau and J-D. Tissot, among other) will contribute to a deeper understanding of RBC homeostasis as a key, informative, and connective piece of the systemic, holistic picture.

To start with, release of extracellular vesicles (EV) is an integral part of reticulocyte and mature RBC development, aging, and stress response. Thangaraju et al. [1] submitted a review article that focuses on the biogenesis, features, and biological activities of RBC-derived EVs (R-EV). Several studies reported that R-EV biogenesis is regulated by variations in the lateral (raft) and vertical (lipid asymmetry) organization of the membrane, as well as by oxidative and calcium stresses, and it seems to be partially distinct compared to the vesiculation of nucleated cells. Some fascinating findings are the presence of ESCRT proteins in the mature RBC proteome and the enrichment of R-EVs in redox homeostasis enzymes. There is evidence that the R-EVs are involved in the disposal of redundant, damaged and/or potentially dangerous material, giving thus, the “kiss of life” to the otherwise moribund cells of origin. Their composition opens a window to putative upstream processes (such as Hb oxidation) and might be downstream related to immunomodulation, inflammation and mainly, coagulation activities as suggested by studies on RBC storage and hematological diseases. Basic underlying mechanisms of such actions involve NO signaling, redox balance in recipient cells, and heme-mediated endothelial dysfunction. R-EV are increasingly recognized as critical players in the dysregulated hemostasis associated with several diseases. The great size of the related literature is indicative of the intrinsic interest of the scientific community in R-EVs and their functions, though the widespread inconsistence in the reported findings suggests inadequate understanding of EV physiology, as well as heterogeneity among (or even unsuitability of) the methodological approaches used. Despite optimistic messages transmitted to the community by the participation of R-EVs in therapeutic schemes, unfortunately, only a limited number of studies included the necessary considerations on risk assessment. Additional roles (commonly studied in platelet-derived or other EVs but less in R-EVs) include intercellular communication and involvement in tissue adaptation to disease and environment. However, there is still a great deal of uncertainty about the possible crosstalk of RBC *per se* with interacting tissues, a currently understudied issue in the field of clinical hematology.

An answer to these issues come by the next review article of Turpin et al. [2] which unravels the thread of intertwined interactions between RBC, platelets, macrophages, and endothelium potentially involved in the development and progression of thrombus and atherosclerotic plaque. It is well established that interactions between PS exposing RBC and platelets, even under flow conditions, can initiate thrombus formation and clotting, via activation of coagulation enzymes and assembly of coagulant complexes. Lysis and phagocytosis of RBC trapped in the proinflammatory and pro-oxidative conditions of the necrotic core of the plaque lead to overheating of macrophages and smooth muscle cells, or release of damage-associated molecule pattern (DAMP) signals (e.g., Hb and iron), respectively. These events have chain reactions towards the following: (i) a further increase in the oxidative stress and inflammation, which contribute to the expansion of the necrotic core of the plaque; (ii) the supply of the plaque with RBC membrane-derived cholesterol, which contributes to plaque development; and (iii) an increase in the intake of RBC and free iron by the local macrophages, which contributes to the formation of foam cells, as well as to destabilization and rupture of the plaque. The pro-atherogenic potential of RBC may be reinforced after modification by glycoxidation under hyperglycemic conditions. Glycation of RBC proteins may have substantial effects on redox and calcium homeostasis, pump activity, membrane deformability and integrity as well as in removal signaling, which not only underlie aggregation, adhesion, and clearance processes, including eryptosis, in RBC but also exert a detrimental impact on the vascular endothelium, substantiating significant potential roles for RBC in the vascular pathologies observed in diabetic patients.

Indeed, Hb glycation leads to ROS generation and oxidative defects in RBC that might induce aging, as shown in the D-galactose accelerating RBC aging model. Blat et al. [3] presented a comparative biochemical and functional characterization of intact cells and RBC membranes in control aging animals versus those of the D-galactose model, by spectroscopic approaches (FTIR-ATR and Raman). The model mimic not only variations in plasma components that follow natural aging in mice (e.g., decrease in LDL levels), but also modifications in mechanical and functional features of RBC (e.g., deformability) and in molecular components, including membrane phospholipid content, unsaturation degree, and acyl chain shortening due to lipid peroxidation. On the other side, the D-galactose model of accelerating aging was found not equal to the natural aging counterpart in terms of RBC senescence markers related to the secondary structures of membrane proteins, levels of cholesterol esters, and total esterified lipids. Thus, even though the D-galactose model recapitulates the age-related rheological modifications, the authors gave a note of caution with regards to its application in mechanistic studies of aging, especially in circulation-related diseases.

RBC aging is a key biomedical issue for many reasons, including the efficacy and safety of transfusions. Oxidative defects and changes in the redox and energy metabolism represent the driving forces for the development of storage lesion that is characterized by accelerated aging. Bardyn et al. [4] from the Switzerland RBC study team of Tissot and Prudent evaluated in parallel the protective effects of ascorbic acid, uric acid, trolox and resveratrol antioxidants on oxidatively challenged stored RBC, through monitoring ROS production (by fluorometry) and label-free digital holographic microscopy. Under the experimental conditions of choice, uric acid seemed to be less effective than other antioxidants against ROS accumulation, while some oxidants and high dose of the antioxidant resveratrol yielded morphological modifications in RBC. This combinational assay that provides complementary information through primary fluorescent (indicative of ROS generation) and secondary microscopy readouts is compatible with high-throughput screening and thus, it may be used for the investigation of RBC aging process, the relation between omics variables and morphology, as well as for the identification of anti-stress treatments and molecules as candidate additives in RBC preservative solutions.

Zimna et al. [5] approached another aspect of the RBC storage lesion, namely, the progressive ion leakage defects. Of note, except for the Cl^-^ ions, the ion concentration levels were in general out of the normal values of human plasma in vivo, already after the second week in the cold. Moreover, the ion variation profiles revealed functional disturbances in membrane with storage time (e.g., in ion carriers, transporters, and exchangers, such as band 3). Certain biochemical and morphological RBC features (as assessed by atomic force microscopy) seemed to follow irreversible modifications in ion exchange across membrane. An interesting correlation between the rate of progressive ion changes and RBC of blood group type O was noticed, with group-O RBC being more responsive to environmental changes, and thus, more susceptible to aberrant ion exchange, a property that may affect their performance in oxygen delivery. As expected, older RBC showed increased K+ efflux. The authors concluded that storage duration is a significant contributor to the ion leakage side of RBC storage lesion, that is associated with morphological and functional disturbances related to gas exchange, maintenance of biconcave disk shape, deformability, and response to environmental conditions.

As biological age does not have to be in accordance with chronological age, it is important to find specific hallmarks and biomarkers that could objectively determine the rate of RBC age. To this end, Hadjesfandiari, Khorshidfar and Devine [6] gathered and commented on laboratory and clinical data highlighting the potential effects of donor variability—in genetic or non-genetic features—on the quality of RBC units and transfusion outcomes. Although conclusions are not always consistent between studies the overall picture substantiates that donor matters. Indeed, the quality of stored RBC, the effectiveness (e.g., Hb increment) or adverse clinical effects of transfusion are related to the donor biology in a way that may overpass the effect of storage age. In support of this, several clinical trials, despite their limitations, unanimously record no correlation between storage age and morbidity/mortality rates. Besides collection, processing methods, and storage additive solutions, the lack of correlation with the easily measured clinical phenotypes reflects wide subclinical (or marginally clinical) inter-donor differences in RBC aging, genetics, and lifestyle. Identification of effectors of RBC quality is crucial for the development of storage strategies and their management in blood banks. Donors with G6PD or pyruvate kinase deficiencies or heterozygous carriers of thalassemic or sickle cell mutations constitute a non-negligible percentage of donor population, at least in specific geographical regions, who may be undiagnosed. Other states include genetic RBC membrane disorders such as microcytic hereditary spherocytosis, familial pseudohyperkalemia (which leads to excessive potassium leakage from RBC), and hereditary hemochromatosis that is characterized by iron overload due to defects in the regulatory hormone hepcidin. Despite evidence pointing out to potential impact of these RBC modifications on storage efficacy and effects, it is not enough to justify recommendations for the blood banks and transfusionists. RBC may further differ as a function of donor sex, age, ethnicity, smoking, alcohol consumption, obesity, medications, and finally, the frequency of donations. A so multifactorial situation of intertwining possible effectors is unlikely to be predicted at individual donor level by a single biomarker, and even if this would be happened, the significant role of recipient variation could drastically change the landscape of outcomes. Systematic recording of donor and recipient data, monitoring of transfusion effects and assessment of both parts by omics technologies would lead to the identification of biomarker panels and profiles able to ensure optimum transfusion therapies at individual base.

In a strongly related research article on donor variation issue, Tzounakas et al. [7] provided further insight into the physiology and aging of RBC from beta thalassemia trait donors. By using electron microscopy, classic biochemical, and contemporary proteomics approaches, they examined whether the morphology and proteome of those RBC are in line with the recently reported better storability and nitrogen/purine metabolism profiles. They revealed better shape preservation in heterozygous than in average control donors’ RBC throughout the storage period. The RBC membrane proteome was characterized by beta thalassemia signatures as well as by novel proteomic features functionally connected to resistance to malaria infection, as reflected in the different membrane levels of surface antigens, stress response proteins (molecular chaperones, proteasome), kinases, transporters, and redox components. In line with the lower RBC fragility and special metabolic characteristics of this donor subpopulation, membrane levels of myosin and arginase were found upregulated as opposed to the downregulated levels of transporters involved in nitrogen, purine, and amino acid metabolism. Storage conditions either balance or augment the thalassemia-related proteomic variations or produce new ones, including excess of skeletal proteins. Biological network analysis revealed novel connections between extracellular vesicles phenotypes and proteasome activity or hemolysis metrics in stored RBC.

RBC responses to pharmaceuticals gain increasingly attention since they highlight RBC contributions to the efficacy of therapies. In a very interesting study submitted by Jani et al. [8] from Pedro Cabrale’s group, it is shown that the pleiotropic anticancer agent RRx-001 (being currently in Phase III trials) leads to preferential RBC localization to tumors and subsequent decrease in tumor viability, via PS expression secondary to Hb oxidation and NO production. The PS^+^ treated RBC aggregate in tumors implanted into animal models following adherence to tumor endothelium in cases it expresses increased number of PS receptors, as demonstrated under cancer microenvironment mimicking conditions of inflammation and hypoxia in vitro. Of note, RRx-001-treated RBC are more cytotoxic to tumor cells compared to the anticancer agent alone, supporting the hypothesis that a stress response of RBC to RRx-001 renders RBC critical effectors of RRx-001 cytotoxicity in tumors.

Specific molecular alterations that RBC undergo in uremic milieu may be also clinically relevant with respect to NO bioavailability issues observed in chronic kidney disease. Among these, a compensatory increase of RBC-NO synthase activation is included. In their article Palmerini et al. [9] reported the impact of renal disease stage and therapeutic strategy (conservative therapy in stage 3 or 4 patients, peritoneal dialysis or hemodialysis in stage 5 patients) on the synthetic NO pathway activity in RBC from uremic patients. They found that the typical three therapeutic approaches affect at a variable way the ATPase activity of the cyclic guanosine monophosphate (cGMP, a biological effector of NO) transporter located on the RBC membrane, leading to lower efflux, and thus to higher cGMP accumulation inside RBC in conservative therapy and hemodialysis compared to the peritoneal dialysis. This finding, in association with variation in plasma markers of NO homeostasis and clinical markers of vascular damage prompt the authors to suggest that the continuous dialysis modality impairs RBC and vascular functionalities to a lesser extent compared to other therapeutic strategies. In the case of peritoneal dialysis, the implications might be modulated by lower levels of nitro-oxidative stress that result by the daily purification of the uremic plasma, as well as by better preservation of residual kidney function and clearance performance in peritoneal dialysis compared to the hemodialysis group, as revealed by the control plasma levels of cGMP in this cohort of stage 5 patients.

Finally, changes in RBC integrity and deformability may affect blood physiology in a systemic way. Low deformability, for instance, has chain effects on blood viscosity and flow as well as on oxygen delivery, and thus on cardiovascular strain and exercise performances. Several studies have focused on these modifications in the blood of athletes after maximal or long-distance running under various climate and altitude conditions. A different RBC phenotype have been described between shorter and prolonged efforts, the later having become increasingly popular. In this context, Robert et al. [10] reported significant differences in the rheological properties and markers of RBC senescence and damage in athletes of long and very long (40 km and 171 km, respectively) mountain racing. Classic marathon distance was associated with increased blood viscosity, RBC aggregation and senescent markers (PS exposure, low CD47, but control plasma microparticles concentration), as opposed to the ultramarathon race, which resulted in decreased blood viscosity and hematocrit, with worse levels of plasma inflammatory factors and of (the probably interrelated) RBC deformability and RBC-membrane exovesiculation. The authors supposed that low blood viscosity after ultra-marathon might facilitate blood flow to muscles and optimize aerobic performance. Notably, neither of these extreme physical exertion conditions led to marked hemolysis or increased ROS accumulation. These findings highlight a variety of conditions underlying the RBC phenotypes and responses to stress, the existence of thresholds, and the effect of systemic factors (e.g., inflammation) on RBC.

In the same context, Nemkov et al. [11] reported metabolic, rheological, and biophysical changes in RBC from well-trained males following a 30 min, high intensity cycling test that resulted in increased circulation, oxygen demand, and increased on- and offloading. The major physiological changes, namely, loss of deformability, increased RBC aggregation, and RBC membrane exovesiculation (but not PS exposure or eryptosis) were closely associated with metabolomic and lipidomic variations predominantly in components involved in oxidative stress response, energy metabolism, and membrane remodeling and repair (including free fatty acids and acylcarnitines). Thus, it seemed that the exercise-induced shear and/or oxidative stresses experienced by RBC induce membrane damage that requires activation of repair and remodeling mechanisms as well as shedding of damaged components and membrane patches in the form of microvesicles. The study highlighted the significance of inter-subject variability over the same power output gradient and identified metabolic pathways and components which are associated with RBC deformability (e.g., acylcarnitines) and microvesicle generation (e.g., CoA precursors). Moreover, it suggests that the metabolic status of RBC could be associated with endurance capacity, and that persistent exercise could result in accelerated RBC aging and loss of functional capability, leading to removal from circulation. This could trigger erythropoiesis to generate younger and more robust RBC, capable of facing oxidant and shear challenges imposed by harsh conditions, related or not to body training. A very interesting and still open question is the potential impact of biological sex and lifestyle on those RBC responses.

Reports of RBC modifications in conditions of systemic oxidant stress, dysregulation of oxidative metabolism, and NO/iron homeostasis from G6PD deficiency and beta thalassemia trait to cancer, from diabetes to cardiovascular and renal diseases and from in vivo aging to the hypothermic storage of blood and exercise performance, simply reflect the centrality of RBC and its secretome in our biological universe, which is based on unique RBC features of communicational effectiveness and functional complexity. We fully recommend this Special Issue of highly scientific value articles prepared by experts in the field to International Journal of Molecular Sciences readers interested in RBC biology.
